# Palliative care initiation in pediatric oncology patients: A systematic review

**DOI:** 10.1002/cam4.1907

**Published:** 2018-12-07

**Authors:** Brian T. Cheng, Michael Rost, Eva De Clercq, Louisa Arnold, Bernice S. Elger, Tenzin Wangmo

**Affiliations:** ^1^ Department of Hematology and Oncology Northwestern University Feinberg School of Medicine Chicago Illinois; ^2^ Institute for Biomedical Ethics University of Basel Basel Switzerland; ^3^ Institute of Psychology Friedrich‐Schiller‐University of Jena Jena Germany

**Keywords:** access, cancer, palliative, pediatric oncology, timing

## Abstract

Palliative care (PC) aims to improve quality of life for patients and their families. The World Health Organization and American Academy of Pediatrics recommend that PC starts at diagnosis for children with cancer. This systematic review describes studies that reported PC timing in the pediatric oncology population. The following databases were searched: PubMed, Web of Science, CINAHL, and PsycInfo databases. Studies that reported time of PC initiation were independently screened and reviewed by 2 researchers. Studies describing pilot initiatives, published prior to 1998, not written in English, or providing no empirical time information on PC were excluded. Extracted data included sample characteristics and timing of PC discussion and initiation. Of 1120 identified citations, 16 articles met the inclusion criteria and comprised the study cohort. Overall, 54.5% of pediatric oncology patients received any palliative service prior to death. Data revealed PC discussion does not occur until late in the illness trajectory, and PC does not begin until close to time of death. Despite efforts to spur earlier initiation, many pediatric oncology patients do not receive any palliative care service, and those who do, predominantly receive it near the time of death. Delays occur both at first PC discussion and at PC initiation. Efforts for early PC integration must recognize the complex determinants of PC utilization across the illness timeline.

## Abstract 2

This systematic review examines the timing of palliative care initiation in pediatric oncology patients to assess the state of palliative care integration. Many pediatric oncology patients do not receive any palliative service, and those who do, typically receive it late in the illness trajectory.

## 1 INTRODUCTION

The World Health Organization (WHO) released a seminal report, titled *Cancer Pain Relief and Palliative Care in Children*, in which it recommended that palliative care (PC) for children with cancer ought to begin at diagnosis, irrespective of prognosis.^1^ Other international health organizations—the American Academy of Pediatrics (AAP),^2^ Institute of Medicine (IOM)^3^, European Association for Palliative Care (EAPC),^4^ and the Royal College of Paediatrics and Child Health (RCPCH)^5^—have all since adopted a similar recommendation.^6^ These calls for earlier PC are grounded in evidence of an unmet need: the high illness burden and degree of suffering are well established among children with cancer.^7^ Additionally, numerous studies have demonstrated that the tight prognostic limits of hospice—a type of PC reserved for the end‐of‐life—are incompatible with the full spectrum of physical, psychological, social, and spiritual needs.^8^, ^9^, ^10^ PC presents an effective solution as both children with cancer and their parents report significantly enhanced quality of life from PC involvement.^11^, ^12^

With the improvements in medical therapy for pediatric oncology patients,^13^ children now survive for longer periods and require extended PC, making pediatric PC an increasingly important area of research.^14^, ^15^ Recent studies have investigated related ethical issues: how and when children should be involved in decision‐making^16^, ^17^, ^18^, ^19^ and what disparities exist in PC access.^20^, ^21^ Moreover, various studies have demonstrated PC for children with cancer is initiated late in the illness trajectory,^22^, ^23^, ^24^ indicating a discrepancy between the normative recommendation for early integration and referral practices in pediatric oncology. Yet, no systematic review of the timing of PC initiation has been conducted to compile this growing body of literature.

Understanding the current state of PC timing is necessary to inform efforts to expand PC access, increase the time that children benefit from PC, and better support pediatric oncologists. As such, the purpose of this study was to systematically review literature describing the current timing practices of PC initiation in children with cancer. The two key events involved in the start of PC are the initial discussion with or without specialist consultation and the first instance of palliative service provided. If PC services started close to time of death, it is important to know if discussion occurred early and PC was deemed unnecessary, or if discussion also occurred late. Knowing the specific timing of these events will identify where in the care continuum barriers may lie. Thus, this review sought to answer a guiding questions: a) what time elapses between cancer diagnosis and PC discussion or consult; b) how long before death does PC discussion occur; c) what is the PC duration received before death, and d) what proportion of children receive PC.

## 2 METHODS

### 2.1 Search methodology

This systematic review of literature on the timing of PC in pediatric oncology patients was performed in accordance with the Preferred Reporting Items for Systematic reviews and Meta‐Analysis (PRISMA) guidelines.^25^ Details of the systematic review protocol were registered on the PROSPERO International Prospective Register of Systematic Reviews (CRD42018108557). We searched PubMed, Web of Science, CINAHL, and PsycInfo for publications between 1 January 1998 and 15 December 2017, examined the citations of included articles for relevant additions, and solicited additional citations through discussion with topic experts. The year 1998 was selected as the beginning date because the WHO declared in this year that PC for children with cancer ought to begin at the time of diagnosis.^1^ The WHO declaration altered the paradigm of PC initiation, so practices prior to this date may not be comparable. We developed the following Boolean search phrase based on controlled vocabulary results from the Medical Subject Headings (MeSH) terms database: (Palliative OR Hospice OR End‐of‐Life) AND (Pediatric* OR Child* OR Adolescent* OR Teen*) AND (Cancer OR Oncology OR Tumor* OR Neoplas*) AND (Duration OR Start* OR Time death OR Timing death OR Begin OR Began OR Time referr* OR Timing referr*). The terms “hospice” and “end‐of‐life” care were included in our search because researchers and healthcare staff commonly equate them with PC, but care was taken during screening and full‐text review to ensure the reported data reflected the first iteration of PC. If the patients in the included study had not previously received PC, then that study was included because the described hospice or EOL care also represented the first time PC was provided. Our search yielded a total of 1220 titles and abstracts across all 4 databases (Table 1).

**Table 1:** Search terms and search results on timing of pediatric palliative care

No.	Search terms	Matches			
		PubMed	CINAHL	PsycINFO	Web of Science
1	Palliative OR Hospice OR End of Life	121 923	41 833	28 926	136 362
2	Pediatric* OR Child* OR Adolescent* OR Teen*	2 104 073	414 163	631 834	1 529 829
3	Cancer OR Oncology OR Tumor* OR Neoplas*	2 585 401	268 727	76 791	2 552 665
4	Duration OR Start* OR Time death OR Timing death OR Begin OR Began OR Time referr* OR Timing referr*	822 311	103 473	185 668	1 810 823
5	1 And 2 And 3 And 4	704	55	81	380

Date of last search: 12th of August 2018

### 2.2 Exclusion criteria

We defined the following a priori exclusion criteria: a) results published after the 1998 starting date of our search, but based on data collected from patients prior to the 1998 WHO recommendation; b) neonates because the type, presentation, and management of neonate cancer differs from those of older children,^19^ c) case studies because these describe exceptional medical situations and would skew our review; d) pilot initiatives that report results of a focused trial to encourage earlier PC consultation without pretrial data; e) no empirical data relevant to at least one of the primary study questions; and f) articles written in a language other than English.

### 2.3 Search results and data extraction

**Figure 1:**
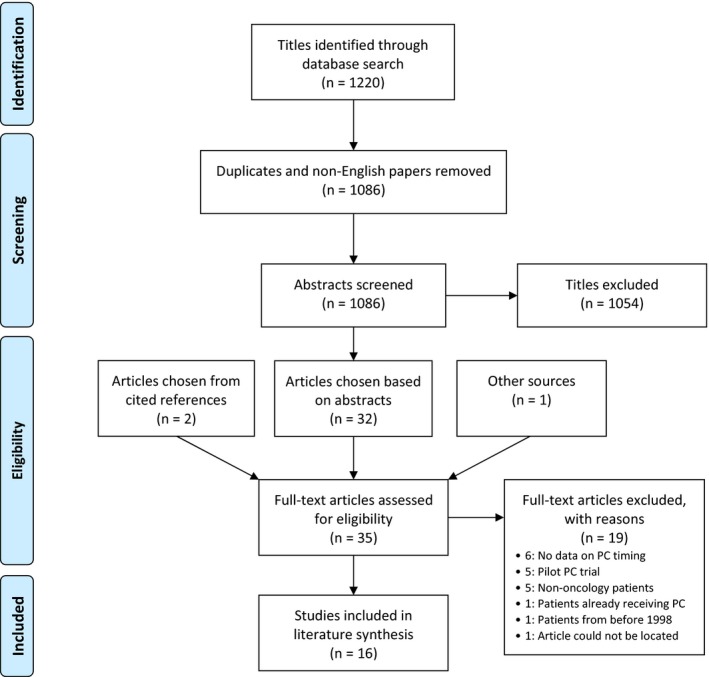
PRISMA flowchart for inclusion of studies

After duplicates were eliminated, two reviewers independently screened the article titles and abstracts to identify relevant articles. Of the 1137 unique citations identified, 1086 were published in English; titles and abstracts were independently screened by two authors, and 31 were selected for full‐text review, in addition to three articles identified from reference lists and expert consultation; 16 remained after full‐text review and comprised the full study cohort (Figure 1).

The authors independently collected the necessary data using a purpose‐built Microsoft Excel extraction form: publication information, sample characteristics, and timing information from each of the articles. The two sets of extracted data were compared to validate the accuracy, and disagreements were resolved by discussion and input of a third investigator.

### 2.4 Analysis

All evaluated outcomes were included in meta‐analysis, including three timeframes—time from diagnosis to first PC discussion, first discussion to death, and duration of PC provided—and proportion who received PC. A study was included in meta‐analysis if it reported mean with standard deviation or median with arithmetic range or interquartile range (IQR). For studies that only reported median and range, mean and standard deviation were calculated based on algorithms presented by Hozo et al^26^ All time data were converted into days. Data integration was conducted using Hedges‐Olkin weighting models for inverse variance.^27^ Random effect models were fitted by applying restricted maximum likelihood estimation, and Forest plots were constructed to visualize the results. All statistical procedures were executed with the metafor package in R‐statistical software.^28^ In cases of significant homogeneity and sufficient number of studies, Lipsey‐Wilson moderator analyses were performed to examine study (sample country [US/non‐US], publication year, terminology [PC‐only/non‐PC], and sample size) and clinical characteristics (principle diagnostic group [solid tumor/blood cancer]) as potential reasons for variability.^29^ A two‐sided *P*‐value ≤0.05 was considered significant for all analyses.

## 3 RESULTS

### 3.1 Characteristics of studies describing PC initiation

Of the 1137 identified studies, 16 studies were included in our review. Mean sample size was 237.2 (standard deviation = 294.5) patients and ranged from 17 to 1208 patients. Included data describes 3796 pediatric cancer patients, including 1438 solid tumor and 1231 hematologic cancer patients. Fifteen articles performed a retrospective medical review of records to study the timing of PC initiation, and one study was a prospective observation across six institutions (Table 2).^30^ Metrics in eight studies were representative of the pediatric oncology population, while the remaining examined subsets of this target population. Timing was a primary outcome in all 16 studies. While all studies used the phrase “palliative care,” it is important to note that six studies also used end‐of‐life (k = 4) or hospice (k = 2) care interchangeably with palliative care.

**Table 2:** Study characteristics of publications included in review (n = 16)

Year	Authors	Country of data collection	Study method	Sample size	Represents population	Terms used for palliative care	Study period
2002	De Graves et al^32^	Australia	Retrospective review	17	Yes	Palliative care	1999‐1999
2005	Bradshaw et al^64^	USA	Retrospective review	145	Yes	End of life	2000‐2001
2008	Menon et al^65^	Malaysia	Retrospective review	247	No	Palliative care	2001‐2007
2011	Feudtner et al^30^	USA & Canada	Prospective data collection	102^a^	Yes	Palliative care	2008‐2008
2011	Tzuh‐Tang et al^66^	Taiwan	Retrospective review	1208	No	Hospice	2001‐2006
2012	Johnston et al^23^	Canada	Retrospective review	273	Yes	Palliative care	2006‐2009
2013	Jalmsell et al^22^	Sweden	Retrospective review	95	Yes	End of life	2007‐2009
2013	Thienprayoon et al^67^	USA	Retrospective review	114	No	Hospice	2006‐2010
2014	Vallero et al^68^	Italy	Retrospective review	39	No	Palliative therapy	2005‐2011
2015	Levine et al^33^	USA	Retrospective review	277	No	End of life	2001‐2005
2015	Vern‐Gross et al^34^	USA	Retrospective review	134	Yes	Palliative care	2001‐2005
2016	Levine et al^69^	USA	Retrospective review	615	No	Palliative care	2007‐2014
2016	Ullrich et al^31^	USA	Retrospective review	147	No	Palliative care	2004‐2012
2017	Ananth et al^70^	USA	Retrospective review	125	Yes	Palliative care	2010‐2014
2017	Hoell et al^71^	Germany	Retrospective review	65	No	End of life	2009‐2016
2018	Rost et al 72	Switzerland	Retrospective review	193	Yes	Palliative care	2008‐2014

Representation of the target population is defined as whether the metrics reported by the study estimate the pediatric oncology population.

a Number of cancer patients isolated from n = 515 cohort.

The 16 included studies were published between 2002 and 2018. The median publication year was 2014, indicating that half of the studies were published in the last 4 years. Most studies (k = 9) reported timing of PC in North America, four in Europe, two in Asia, and one in Australia. However, there was little diversity in World Bank income classification: 15 included from high‐income countries and one from an upper‐middle‐income country.

### 3.2 Time from diagnosis to PC discussion

**Figure 2:**
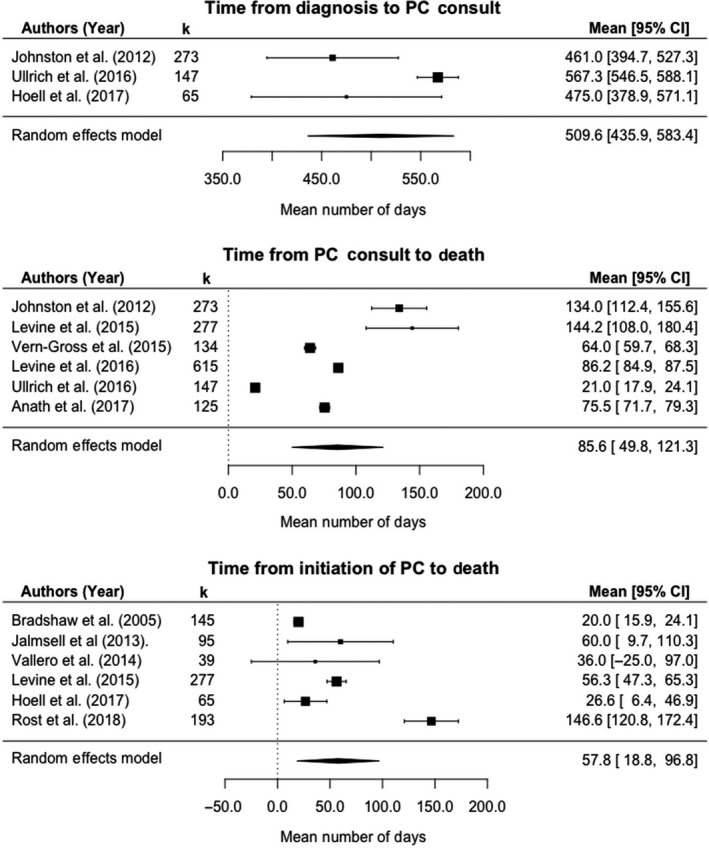
Timing and duration of palliative care provided to children with cancer

Time from diagnosis to first PC discussion was reported in three studies, which included 485 pediatric oncology patients (Figure 2). In our random effects model, the weighted mean time to PC consult was 509.6 (standard error (SE) [95% confidence interval (CI)]: 37.6 [435.9‐583.4]) days. PC discussion did not occur at diagnosis as recommended by WHO and AAP guidelines (*P *< 0.0001). Test for heterogeneity suggested large variation in effect sizes within these studies (Q[2] = 11.7, *P* = 0.003), and Higgins *I* ^2^ statistic demonstrated 78.9% of variability is not attributable to sampling error. There was an insufficient number of studies for moderator analysis.

### 3.3 Time from PC discussion to death

Six studies, including 1571 patients, reported time from PC discussion to death with a weighted mean length of 85.6 (SE [95% CI]: 18.3 [49.8‐121.3]) days. Of note, this number is significantly smaller than the mean time from diagnosis to PC discussion, indicating that PC is discussed late in the illness timeline. An additional study reported median time was less than 37 days, but this study could not be included in our descriptive analysis because it did not report a measure of variation.^30^ In the two studies that reported both time from diagnosis to PC discussion and PC discussion to death, time from PC discussion to death was comparatively short, comprising 25.4% and 3.6% of the total illness duration.^23^, ^31^ Heterogeneity was significant for this outcome (Q[5] = 1547.6, *P *< 0.0001), and Higgins I^2^ statistic was 99.8%, suggesting much of the variability across studies was due to the heterogeneity. However, the role of individual moderators in this variation could not be investigated due to the small number of studies.

### 3.4 Duration of PC

Time from formal PC initiation to death was reported in six studies, including 814 patients. One additional study reported a mean PC duration of 69.4 days, but did not report a measure of variation and could not be integrated in our duration model.^32^ In the random effects model for PC duration, mean duration was 57.8 (SE [95% CI]: 19.9 [18.80‐96.8]) days. Two studies quantified both time from PC discussion to death and PC duration: Levine et al reported 144.2 and 56.3 days, respectively, while Vern‐Gross and colleagues stated 64 and 31 days.^33^, ^34^ Taken together, these findings reveal initial PC discussion does not often result in prompt PC initiation. Effect sizes varied across studies (Q[5] = 134.8, *P *< 0.0001), and 97.8% of variability came from a source other than sampling error. Again, moderator analyses could not be conducted due to the small number of studies.

### 3.5 Proportion with PC

The weighted mean percentage of patients who received PC prior to death was 54.5% (SE [95% CI]: 8.2% [38.5%‐70.5%]) across 12 studies and 3467 patients. Higgins *I* ^2^ statistic was 99.2%; test of heterogeneity was significant (Q[11] = 2230, *P *< 0.0001), and moderator analysis was conducted to investigate the effects of methodological and sample characteristics.

**Figure 3:**
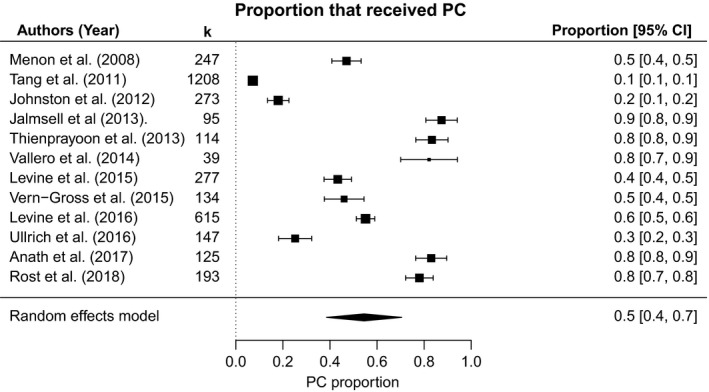
Proportion receiving palliative care in children with cancer

In mixed‐effects models for moderator variables, increasing sample size was associated with a decline in proportion receiving PC (Q[1] = 6.1, *P* = 0.01). Mean PC proportions in US vs non‐US studies were 56.0 vs 53.0 (Q[1] = 0.03, *P* = 0.86); PC proportion has not changed significantly over time (Q[1] = 1.4, *P* = 0.24). Studies exclusively using the PC terminology reported a mean provision fraction of 54.2%, compared to 55.1% in studies that also used EOL or hospice terminology (Q[1] = 0.003, *P* = 0.96). In subgroup analysis by malignancy type, weighted proportion was 39.9% (95% CI: 12.0%‐67.9%) in studies with predominantly hematological malignancies, 54.7% (29.7%‐79.6%) in those with mostly solid tumor patients, and 66.5% (27.1%‐100.0%) for studies in which principle cancer type could not be identified. Despite the variation, cancer type was not significant on moderator analysis (Q[2] = 1.3, *P* = 0.53) (Figure 3).

## 4 DISCUSSION

This systematic review summarizes the timing of PC services for pediatric oncology patients reported in 16 publications published between 2002 and 2018. No systematic review has been conducted on the timing of PC initiation for children with cancer, and the present study is the first to compile the growing literature on pediatric oncology PC practices. Comparing PC provision within studies and across publications, it is apparent that PC integration was delayed at two time points: first PC discussion occurs late in the illness trajectory, and there is a delay between the initial conversation and start of PC. The growing number of publications and pilot initiatives demonstrate genuine energy to embed PC into conventional healthcare and improve the timing of PC discussion.^33^, ^35^, ^36^, ^37^ However, the results of this study suggest that PC integration at diagnosis remains an unmet objective.

Our finding that only 54.5% of pediatric oncology patients received any PC before death may suggest there are structural barriers that inhibit availability of PC. A multinational review of Children's Oncology Group institutions, which serve more than 90% of pediatric oncology patients in the US, found only 60% of providers offer PC services.^38^ Potential reasons for the absence of PC services include lack of coordination between oncology providers and palliative programs outside the hospital, restrictive reimbursement models, and ambiguous roles of members in the care team.^39^ While previous studies established the deficiencies in hospital infrastructure and our results demonstrate the effects on timing of PC provision, the drivers that cause hospitals to offer pediatric PC are not well understood. Further research is recommended to identify socioeconomic and geographic disparities associated with lower pediatric PC provision. Such research would identify areas where PC is underutilized in children with cancer and guide interventions to increase PC provision.

Another proposed explanation in literature for the lack of timely PC provision is the shortage of clinicians capable to deliver PC to children with cancer.^40^ In a survey of PC providers—physicians, nurses, and other staff members—one‐third of respondents cited insufficient training in PC as a barrier to earlier PC integration.^41^ The limited exposure to targeted PC education in residency and fellowship means hospitals do not have staff with the training to meet the palliative needs of pediatric oncology patients.^42^, ^43^, ^44^ Improvements have been made to increase training opportunities as the number of pediatric PC fellowships in the US have doubled since 2013.^45^ However, adequate workforce availability remains a priority to expand the number of institutions that can offer PC to children with cancer.

Training in PC principles is not important solely for designated PC staff; communication by the primary oncologist has a critical role in initiating the first PC discussion with the patient and family. Physicians recognize the child's poor prognosis, on average, almost twice as early as parents do and thus, are often given the task to communicate the bad news to parents, explain treatment complications, maintain hope, and calibrate parental expectations.^46^ Physicians, however, do not consistently articulate this prognosis effectively to the family of children with cancer: parents are more likely than their treating oncologist to indicate the primary goal is cure,^47^ and 61% of parents were more optimistic about their child's odds of a cure than the physician was.^15^ Physicians view broaching PC as a stressor and delay the conversation, focusing instead on treatment arrangements.^48^, ^49^

Formal training is associated with feeling comfortable to manage end‐of‐life issues, but 75% of pediatric oncologists have not had any formal end‐of‐life training.^50^ This communication deficit may cause parents to be overly optimistic and encourage them to pursue aggressive treatment until the physician is certain of their child's imminent death.^47^ Communication skills trainings, supporting resources, and a team‐based approach have shown promise as a means to facilitate earlier advance care planning and PC referral.^51^, ^52^

Multiple studies have cited conceptual confusion between PC and hospice or EOL care, as well as the stigma of hopelessness associated with PC, as significant obstacles to early PC discussions.^6^, ^53^, ^54^, ^55^, ^56^ The International Classification of Disease coding system considers hospice and end‐of‐life care as synonymous with palliative care, and US clinicians are instructed to bill these services identically.^57^ There are even definitional inconsistencies between and among PC guidelines of what palliative care constitutes.^58^ If parents equate PC with EOL support, then it is understandable they will oppose the suggestion of PC at diagnosis or soon after. Yet, the Patient Protection and Affordable Care Act Section 2302 “Concurrent Care for Children” recognizes the difference between PC and EOL care and guarantees coverage under Medicaid or the Children's Health Insurance Program for concurrent hospice and curative care for all children under age 21.^59^ Adoption of a new phrase without the same connotations of death may support physicians in patient discussions. While the use of new terminology in pediatric oncology has not been tested, a survey found that compared to “supportive care,” the phrase “palliative care” was associated with decreased hope and increased distress in adult oncology patients and their families.^60^ Furthermore, in a survey of 646 Canadian physicians, pediatric oncologists reported they would refer patients earlier if PC was renamed “supportive care.”^61^ Additional research is warranted to devise methods to encourage earlier PC in the pediatric setting.

In moderator analysis, larger studies reported lower rates of PC utilization, suggesting that small single‐institution studies may overestimate PC provision. Future research on the topic should consider sample size and setting when designing studies. Literature in which hospice or EOL terminology was used did not vary in results when compared to studies that only used PC terminology; this indicates that despite the connotational significance of terminology in the clinical setting, researchers frequently use these phrases interchangeably. We recommend that researchers use caution to avoid confusion in reporting results and communicating findings to clinicians.

Finally, US location, temporal trend, and cancer type were not moderators of PC utilization. However, there was notable difference in rates between blood cancer and solid tumor. Pediatric blood cancers tend to have higher survival rates than solid tumors,^62^ which raises concerns that hematological cancer patients may receive more aggressive curative therapy until close to time of death. The low number of studies may explain why cancer type was not a significant co‐variate in our moderator analysis, but additional research is warranted to further investigate a possible difference by malignancy type.

### 4.1 Limitations

Our analyses coalesce data across multiple sites and countries, which increase our confidence that these findings reflect true practice. All included studies were retrospective medical records reviews or prospective collection, which enhances the comparability across studies and reduces the chance of measurement error. Additionally, 12 of our 16 studies had sample sizes with k > 100. As such, we could calculate robust estimations of true effects. There are also limitations to our study. Inherent to systematic reviews, all included data were already published and may be affected by publication bias. Second, studies primarily originated from research in well‐developed regions of the world. Research is needed outside of developed countries to better understand the state of PC access and availability for children with cancer worldwide.

Sample size may partially explain the variation in PC timing observed in our results. Yet, there are other potential moderators that were not reported and could not be tested. Culturally determined understandings of when a condition is considered terminal (eg prognosis smaller than 10% vs 5%) may affect timing in different settings. Other demographic (eg income, ethnicity), clinical (eg comorbid chronic conditions), and methodological (eg different definitions of when PC began) characteristics may also provide valuable context for the observed results and thus, warrant further study. Aside from these limitations, this study is the first systematic review on the timing of PC and sets a foundation for efforts to improve quality of life for children with cancer.

## 5 CONCLUSION

Our results underscore that PC starts too late for children with cancer and are not in line with the recommended AAP, WHO, IOM, EAPC, and RCPCH guidelines.^1^, ^2^, ^4^, ^5^ Palliative care discussion does not occur until far into the illness, and PC does not start until much later. Each case is unique and must be evaluated using the caring physician's best medical judgment and with respect to the patient and family, but holistically, there is much room for improvement regarding PC timing for this patient population. Effective, timely communication maintains patient quality of life and dovetails the transition to palliative care, while poor communication may lead to poor treatment planning and psychological harms for the child and family.^63^ Reasons for delayed discussion and initiation include insufficient resources and infrastructure, lack of training, and negative connotations attached to PC.

Findings in the present study regarding timings of PC discussion and initiation suggest pronounced obstacles across the PC lifecycle. As such, initiatives focused on specific referral points likely will not succeed. Programs designed to target PC timing must be robust and coordinated across the PC lifecycle to achieve effective improvement. Palliative care is central in pediatric oncology, and continued advocacy is necessary to promote optimal care for this patient population.

## CONFLICT OF INTEREST

The authors declare no conflicts of interest.
